# Work incapacity and psychiatric patient care following attempted suicide – a cohort study of 65 097 Swedish twins

**DOI:** 10.1017/S0033291722000435

**Published:** 2023-06

**Authors:** Anton Lindberg, Pia Svedberg, Annina Ropponen, Jurgita Narusyte, Mo Wang

**Affiliations:** 1 Division of Insurance Medicine, Department of Clinical Neuroscience, Karolinska Institutet, Stockholm, Sweden; 2 Finnish Institute of Occupational Health, Helsinki, Finland; 3 Center of Epidemiology and Community Medicine, Stockholm County Council, Stockholm, Sweden

**Keywords:** Disability pension, mental health, sick leave, suicide attempt, twin study

## Abstract

**Background:**

Research is scarce on the role of familial factors and previous psychiatric care on the association between suicide attempt and future work incapacity as well as deterioration in mental health. We aimed to investigate the associations between suicide attempt and sickness absence, disability pension and psychiatric patient care and to study the influence of previous psychiatric care and familial factors (genetics and shared environment) on the associations.

**Methods:**

The study included 65 097 twins living in Sweden on 31st of December 2006, aged 19–60 years. The twins were followed 2007–2013 regarding sickness absence, disability pension, inpatient care or specialized outpatient care for a mental diagnosis. Cox regression models were performed for the whole sample, and conditional models for discordant twin pairs. The analyses were also stratified by psychiatric care before 2007.

**Results:**

We found that suicide attempt predicted sickness absence, disability pension, and future mental diagnosis among the whole sample. The discordant twin pair analyses showed that the association between suicide attempt and sickness absence or disability pension was influenced by familial factors. Stratified analyses of individuals with or without psychiatric care before 2007 showed that previous psychiatric care had some impact on the associations.

**Conclusions:**

A suicide attempt is a risk factor for work incapacity and psychiatric patient care. Familial factors and previous psychiatric care play a role in the associations between attempting suicide and work incapacity as well as psychiatric patient care. These factors are important when developing measures preventing work incapacity among those with a suicide attempt.

## Introduction

Suicidal behavior is associated with poor mental, physical, and social health, hence being a major public health concern (Skegg, 2005). A person's first suicide attempt tends to occur more frequently during adolescence and the likelihood of repetition is about 15% in Western countries (Skegg, 2005). Thus, it is also a highly significant risk factor for subsequent suicidality and mortality. On account of these facts, there has been an understandable interest in suicide prevention and most research to date has been concerned with risk factors for suicidal behavior. Consequently, studies investigating the effects of attempting suicide on subsequent work incapacity and psychiatric patient care have fallen by the wayside. In regard to work incapacity, studies using sickness absence and disability pension as measures of short-term and long-term work incapacity, respectively, are particularly sparse, despite disability and exclusion from the job market being an ever-present cause for concern in the industrialized world (Niederkrotenthaler et al., 2017; OECD, 2010). In the interest of researchers, decision makers and society at large this matter needs to be investigated further, since poor mental health, exclusion from the job market, and dependence on social security benefits have considerable negative effects for individual well-being and on the economy (Harkko, Virtanen & Kouvonen, 2018; Vingård, Alexanderson, & Norlund, 2004).

Work incapacity, psychosocial stressors, restricted educational attainment and lack of social support are known to exacerbate poor health (Hawton, Saunders, & O'Connor, 2012), and previous studies have found an association between sickness absence/disability pension and subsequent suicidal behavior or other morbidities (Mather, Blom, Bergström, & Svedberg, 2019; Rahman, Alexanderson, Jokinen, & Mittendorfer-Rutz, 2016; Wang et al., 2014; Wang, Alexanderson, Runeson, & Mittendorfer-Rutz, 2016). Moreover, since suicidal behavior generally has an early onset and is highly associated with poor mental and somatic health, it is likely to be an important risk factor for subsequent work incapacity and exclusion from the job market, particularly for those with previous mental disorders. Therefore, it is crucial to identify the pathways to work incapacity in people with previous psychiatric care and suicidal behavior in order to diminish the number of individuals who experienced work incapacity and exit from the job market. Currently, few prospective studies have investigated the association between attempted suicide and subsequent work incapacity, but all reported higher incidences of sickness absence and disability pension among youth who attempted suicide (Niederkrotenthaler, Helgesson, Rahman, Wang, & Mittendorfer-Rutz, 2018; Niederkrotenthaler et al., 2014, 2017). These studies were based on Swedish register data and adjusted for sociodemographic factors, psychiatric disorders, and somatic disorders. They were however not able to control for familial factors.

The main benefit of a twin study design compared to population-based studies is the possibility to control for familial factors, i.e. genetic and shared environmental factors (e.g. factors shared by twins when they reared together mainly in childhood including family values, child-rearing practices, divorce, family income etc.) that are shared by the twins in a pair. This enables to estimate the influence of genetics and shared environmental factors on the associations investigated by comparing monozygotic (MZ) twins and dizygotic (DZ) twins with results of the entire cohort of twins (Røysamb & Tambs, 2016). MZ twin pairs are assumed to be genetically identical and, in most cases, have a shared family environment. Therefore, any difference in outcomes between MZ co-twins can be assumed to be due to non-shared environmental factors. DZ twin pairs on the other hand share on average 50% of genetics and 100% of shared family environment, like any other pair of siblings. Differences in outcomes within a DZ twin pair can therefore be both due to genetics and a non-shared environment.

Previous twin studies have shown that genetics explain about 36% of the total variance in sickness absence (Seglem et al., 2020; Svedberg, Ropponen, Alexanderson, Lichtenstein, & Narusyte, 2012) and about 49% of the total variance in disability pension (Narusyte et al., 2011). Furthermore, the corresponding estimates for different anxiety disorders are between 32% and 43%, and for major depressive disorder 38% (Hettema, Neale, & Kendler, 2001; Kendler, Gatz, Gardner, & Pedersen, 2006). A recent study also reported that genetic factors are a risk factor for suicidal behavior (Hirvikoski et al., 2020). These studies all highlight the importance of controlling for familial factors when studying the associations between attempted suicide and subsequent work incapacity and psychiatric patient care, as familial factors have a significant effect and can otherwise confound the results.

## Aims

The aim of this study was to investigate the associations between suicide attempt and subsequent work incapacity and psychiatric patient care (i.e. inpatient care and specialized outpatient care due to a mental diagnosis) and to assess to what extend the associations were influenced by familial factors and previous psychiatric care.

## Methods

### Data sources

The present study was based on data from the Swedish Twin project of Disability pension and Sickness absence (STODS), which include data from several sources. A total of 119 907 twin individuals born between 1925 and 1990 were identified from the Swedish Twin Registry which includes nearly every twin born in Sweden (Lichtenstein et al., 2002). Data on sociodemographic variables were available through the Longitudinal Integration Database for Health Insurance and Labour Market Studies (LISA) from Statistics Sweden (Ludvigsson et al., 2011). Information about sickness absence and disability pension was gathered from the MicroData for Analyses of Social insurance (MiDAS) register handled by the Swedish Social Insurance Agency. Data were also collected from the Swedish National Patient Register (date and diagnosis for inpatient care and specialized outpatient care) (Ludvigsson et al., 2011), Prescribed Drug Register (date of prescription of antidepressants, anxiolytics, and hypnotics and sedatives), and Cause of Death Register (death date), all these registers are held by the National Board of Health and Welfare. The data were pseudonymized by replacing personal identity numbers with unrelated ID numbers by Statistics Sweden before being handled by researchers.

### Sample and data

**Fig. 1.**
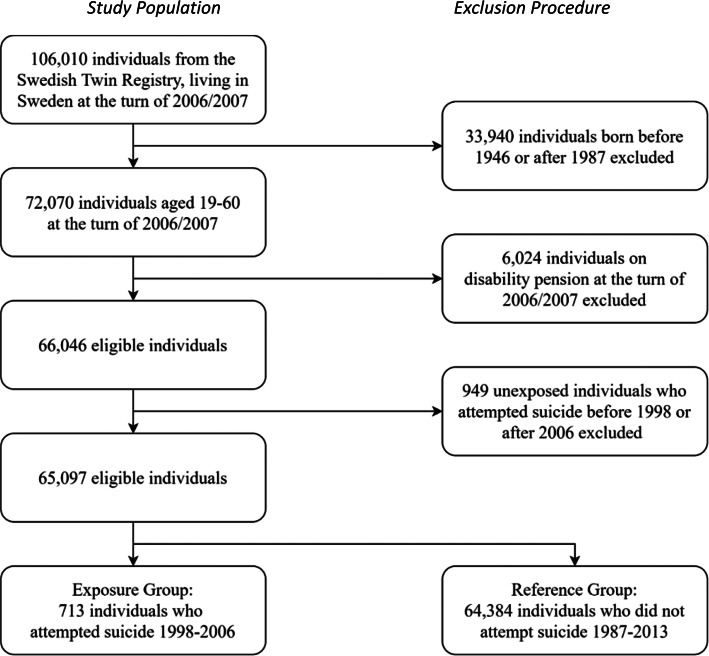
Flow-chart of the study population.

This prospective twin cohort study utilized register data from a population-based twin cohort with all Swedish twins living in Sweden at 31st December 2006 (*n* *=* 106 010). First, 33 940 individuals under the age of 19 or over the age of 60 at 31st December 2006 were excluded from the study. These individuals were assumed to have a lower risk of sickness absence or disability pension during follow-up time due to their age. Second, 6024 individuals who were already on disability pension during the start of the follow-up period were excluded from the study. Moreover, 949 individuals who did not attempt suicide in 1998–2006 but did attempt suicide outside this time period were also excluded from the study. This led to that 65 097 individuals remained after these exclusions, of which 713 were exposed to suicide attempt in 1998–2006 while the remaining 64 384 individuals comprised the reference group who didn't have any attempted suicide in 1987–2013 (Fig. 1). Among them, 57 086 were part of a complete twin pair whereas 8011 were single individuals, 16 923 were same-sex DZ twins, 21 110 opposite-sex DZ twins, 16 259 MZ twins, and 10 805 were of unknown zygosity.

### Exposures

Individuals who received either inpatient care in 1998–2006 or specialized outpatient care in 2001–2006 due to attempted suicide were considered exposed. The time frames between inpatient care and specialized outpatient care differed due to data on specialized outpatient care being unavailable before 2001. The reference group comprised of individuals fitting the same inclusion criteria, but without any inpatient care or specialized outpatient care due to attempted suicide in 1987–2013. Attempted suicide was operationalized as the diagnostic codes for both intentional self-harm (X60–X84) and event of undetermined intent (Y10–Y34) according to the International Classification of Diseases, 10th revision (ICD-10).

### Social insurance in Sweden

Sickness absence benefits are available to everyone in Sweden older than 16 with a minimum qualifying annual income, unemployment benefits, or parental benefits. Usually, the first day of sickness absence is considered a qualifying day where no benefits are paid, though self-employed may have more qualifying days depending on their insurance. For employed individuals, benefits are paid by the employer between days 2 and 14, and from day 15 they are paid by the Social Insurance Agency. Disability pension is a form of compensation for longer-lasting or permanent work incapacity due to an injury or disease. It is available to everyone between 19 and 64 years old, though those aged 19–29 can only get temporary disability pension (SIA, 2019).

### Outcomes

The follow-up period was from 2007–2013, with the measured outcomes being:

1. Short-term work incapacity, defined as receiving sickness absence benefits.
2. Long-term work incapacity, defined as receiving disability pension benefits.
3. Psychiatric patient care, defined as receiving inpatient care or specialized outpatient psychiatric care for a mental disorder (diagnostic codes F00–F99 in ICD-10).

### Covariates

Covariates controlled for in the study were sex, age, level of education, area of residence, and family situation in 2006, as well as sickness absence, unemployment, and inpatient care or specialized outpatient care for a common mental disorder or musculoskeletal disorder between 2004 and 2006 and psychiatric medication between 2005 and 2006 (Table 1). Common mental disorders included ICD-10 diagnostic codes F32 (depressive episode), F33 (recurrent depressive disorder), F40 (phobic anxiety disorders), F41 (other anxiety disorders), F42 (obsessive-compulsive disorder), and F43 (reaction to severe stress, and adjustment disorders). Musculoskeletal disorders corresponded to the ICD-10 diagnostic codes M00–99. Psychiatric medication prescribed between the years 2005 and 2006 was also controlled for, namely antidepressants (N06A), anxiolytics (N05B), and hypnotics and sedatives (N05C) according to the Anatomical Therapeutic Chemical (ATC) Classification System. All the covariates were assumed as important factors associated both with exposure and outcomes and they were also used as covariates in other similar studies (Niederkrotenthaler et al., 2018, 2014, 2017).

**Table 1.** Descriptive statistics of the study population with χ^2^ probability values for proportional differences in distribution between the exposure and the reference group

	Study population^a^	Exposure^b^	Reference	*p*
	(*n* = 65 097)	(*n* = 713)	(*n* = 64 384)	
	*n* (%)	*n* (%)	*n* (%)	
Sex				0.009
Women	31 790 (48.8)	383 (53.7)	31 407 (48.8)	
Men	33 307 (51.2)	330 (46.3)	32 977 (51.2)	
Age (years)				<0.001
19–30	18 753 (28.8)	312 (43.8)	18 441 (28.6)	
31–40	15 214 (23.4)	152 (21.3)	15 062 (23.4)	
41–50	15 589 (23.9)	137 (19.2)	15 452 (24.0)	
51–60	15 541 (23.9)	112 (15.7)	15 429 (24.0)	
Zygosity				0.366
Monozygotic	16 259 (25.0)	17 0 (23.8)	16 089 (25.0)	
Dizygotic same sex	16 923 (26.0)	172 (24.1)	16 751 (26.0)	
Dizygotic different sex	21 110 (32.4)	235 (33.0)	20 875 (32.4)	
Unknown	10 805 (16.6)	134 (18.8)	10 555 (16.4)	
Level of education (years)				<0.001
Low (≤9)	7966 (12.2)	160 (22.4)	7806 (12.1)	
Medium (10–12)	34 082 (52.4)	393 (55.1)	33 689 (52.3)	
High (>12)	22 957 (35.3)	156 (21.9)	22 801 (35.4)	
Missing information	92 (0.1)	4 (0.6)	88 (0.1)	
Area of residence^c^				<0.001
Big cities	23 380 (35.9)	200 (28.1)	23 180 (36.0)	
Medium-sized cities	23 482 (36.1)	297 (41.7)	23 185 (36.0)	
Small cities/villages	18 235 (28.0)	216 (30.3)	18 019 (28.0)	
Family situation				<0.001
Married/living with partner without children	10 802 (16.6)	75 (10.5)	10 727 (16.7)	
Married/living with partner with children	20 231 (31.1)	136 (19.1)	20 095 (31.2)	
Single/divorced/separated/widowed without children	28 371 (43.6)	413 (57.9)	27 958 (43.4)	
Single/divorced/separated/widowed with children	2940 (4.5)	54 (7.6)	2886 (4.5)	
Adolescents living with parents, 16–20 years	2753 (4.2)	35 (4.9)	2718 (4.2)	
Work incapacity				
Sickness absence 2004–2006	12 475 (19.2)	255 (35.8)	12 220 (19.0)	<0.001
Unemployment 2004–2006	14 848 (22.8)	293 (41.1)	14 555 (22.6)	<0.001
Health care and medication				
Common mental disorder 2004–2006	1177 (1.8)	150 (21.0)	1027 (1.6)	<0.001
Musculoskeletal disorder 2004–2006	5286 (8.1)	100 (14.0)	5186 (8.1)	<0.001
Psychiatric medication prescribed 2005–2006	6948 (10.7)	258 (36.2)	6690 (10.4)	<0.001
Psychiatric care for a mental disorder before 2007	2945 (4.5)	331 (46.4)	2614 (4.1)	<0.001
Outcomes				
Sickness absence 2007–2013	21 475 (33.0)	338 (47.4)	21 137 (32.8)	<0.001
Disability pension 2007–2013	1438 (2.2)	75 (10.5)	1363 (2.1)	<0.001
Psychiatric patient care 2007–2013	4508 (6.9)	263 (36.9)	4245 (6.6)	<0.001

a Twins aged 19–60 and living in Sweden at the turn of 2006/2007, with no ongoing disability pension and no suicide attempt between 1987 and 1997 or 2007 and 2013.

b Attempted suicide between 1998 and 2006.

c Big cities: Stockholm, Gothenburg, and Malmö; medium-sized cities: more than 90 000 inhabitants within 30 km distance from the city center; small cities/villages: less than 90 000 inhabitants within 30 km distance from the city center.

### Statistical analyses

Proportional differences in the distribution of variables between the exposure and the reference group were assessed with χ^2^ testing. The association between attempted suicide and subsequent work incapacity and psychiatric patient care was measured using Cox proportional hazards regression with stepwise adjustment for covariates. The proportional hazards assumption was tested by examining the log–log curves for each categorical factor, and all the curves were found to be acceptably parallel. Censoring occurred due to emigration, death, or the end of the follow-up period, whichever took place first. Censoring also occurred due to disability pension in the analyses where sickness absence was the measured outcome. Besides the crude model, the Cox models were adjusted for the covariates in the multivariate model. Analyses were stratified by the presence of inpatient care or specialized outpatient psychiatric care for a mental diagnosis (ICD-10 codes: F00–F99) before 2007 to determine if this had an impact on the effect of exposure on the outcomes.

Co-twin control analyses were performed using conditional Cox regression models to account for familial factors. The conditional analyses included both MZ and DZ twin pairs where the twins were discordant for outcomes, i.e. where one of the twins in a pair received sickness absence, disability pension or psychiatric care for a mental disorder in 2007–2013 and the other did not. First, the analyses were run for MZ and DZ twins together to see whether there was any influence of familial factors on the associations between attempted suicide and subsequent work incapacity/psychiatric patient care. If the associations from the whole cohort attenuated or changed direction in the discordant twin pair analyses, this would suggest the presence of familial confounding, that is, shared early environmental factors and/or genetics. Second, analyses stratified by zygosity were run. If an association from the whole cohort was found to be weaker but still remained significant among discordant DZ twin pairs, and was to a larger extent attenuated for MZ twin pairs, this would indicate the presence of genetic confounding (McGue, Osler, & Christensen, 2010). Data processing was performed using SPSS for Windows version 23.0.

## Results

Table 1 contains descriptive statistics of the study population at baseline and during the follow-up. All variables but zygosity varied significantly in proportional distribution between the exposure and the reference group. It was more common for women than men to attempt suicide, making up 48.8% of the study population but 53.7% of the exposure cases (*p* = 0.009). Individuals who attempted suicide had more often sickness absence or unemployment 2004–2006, inpatient care or specialized outpatient care for common mental and musculoskeletal disorders 2004–2006, psychiatric medication prescribed 2005–2006, psychiatric care for a mental diagnosis before 2007, and the outcomes sickness absence, disability pension, or psychiatric care for a mental diagnosis between 2007 and 2013 (*p* < 0.001).

Table 2 shows the HRs for sickness absence, disability pension, or inpatient care or specialized outpatient care for a mental diagnosis between 2007 and 2013, following an attempted suicide between 1998 and 2006. In the crude model, suicide attempt was significantly associated with receiving sickness absence (HR: 1.74, 95% CI 1.56–1.93), disability pension (HR: 5.26, 95% CI 4.17–6.63), and psychiatric care for a mental diagnosis (HR: 7.24, 95% CI 6.39–8.20). The final model included all covariates with the largest reduction in the associations between suicide attempt and sickness absence, disability pension and psychiatric patient care, though all the HRs remained significant in the final model. In the conditional analyses, suicide attempt was still significantly associated with all outcomes for DZ twins. The HRs for suicide attempt were lower in MZ twins compared to DZ twins. For MZ twins the association between suicide attempt and psychiatric patient care was statistically significant (HR: 2.59, 95% CI 1.84–3.64), but no longer for the association between suicide attempt and sickness absence (HR: 0.99, 95% CI 0.69–1.43) or disability pension (HR: 1.67, 95% CI 0.91–3.07).

**Table 2.** Crude and adjusted hazard ratios with 95% confidence intervals for the outcomes between 2007 and 2013, following attempted suicide between 1998 and 2006

	Model 0^a^	Model 1^b^	Model 2^c^	Model 3^d^	Conditional analysis DZ twins	Conditional analysis MZ twins
	HR (95% CI)	HR (95% CI)	HR (95% CI)	HR (95% CI)	HR (95% CI)	HR (95% CI)
Sickness absence	1.74 (1.56–1.93)	1.85 (1.66–2.06)	1.71 (1.54–1.91)	1.24 (1.11–1.38)	1.41 (1.16–1.72)	0.99 (0.69–1.43)
Disability pension	5.26 (4.17–6.63)	6.36 (5.04–8.03)	5.29 (4.18–6.69)	1.81 (1.41–2.32)	2.76 (1.94–3.92)	1.67 (0.91–3.07)
Psychiatric patient care	7.24 (6.39–8.20)	6.69 (5.91–7.58)	6.07 (5.35–6.88)	2.48 (2.17–2.83)	3.33 (2.73–4.06)	2.59 (1.84–3.64)

a Model 0: crude.

b Model 1: adjusted for sex and age.

c Model 2: adjusted for sex, age, level of education, area of residence, and family situation.

d Model 3: adjusted for sex, age, level of education, area of residence, and family situation; sickness absence, unemployment, inpatient care or specialized outpatient care for common mental disorder, and musculoskeletal disorder between 2004 and 2006; psychiatric medication prescribed between 2005 and 2006.

The whole sample was stratified by having received psychiatric care for a mental diagnosis before 2007 and showed significant associations between exposure and the outcomes in both groups, i.e. among those who had received psychiatric care and those which had not. The same pattern remained after adjusting for covariates, except for disability pension (Table 3). The conditional analyses of DZ twins with or without any psychiatric care before 2007 showed significant associations between suicide attempt and sickness absence and disability pension and psychiatric patient care, except for sickness absence (HR: 0.91, 95% CI 0.69–1.21) and care for mental diagnosis (HR: 1.27, 95% CI 0.80–2.02) for individuals without any mental disorder before 2007. Moreover, the HRs for sickness absence and psychiatric patient care were higher in individuals with mental disorders before 2007 compared to those without previous psychiatric care among DZ twins. In MZ twins none of the associations was significant, except for subsequent psychiatric patient care in the group that had received psychiatric care for mental diagnoses before 2007 (HR: 2.90, 95% CI 1.93–4.36).

**Table 3.** Crude and adjusted hazard ratios with 95% confidence intervals for the outcomes between 2007 and 2013, stratified by psychiatric care for a mental disorder before 2007

	Model 0^a^	Model 1^b^	Conditional analysis DZ twins	Conditional analysis MZ twins
	HR (95% CI)	HR (95% CI)	HR (95% CI)	HR (95% CI)
Psychiatric care for a mental disorder before 2007				
Sickness absence	1.44 (1.23–1.68)	1.21 (1.03–1.41)	2.89 (2.16–3.86)	1.42 (0.78–2.58)
Disability pension	2.02 (1.54–2.66)	1.58 (1.19–2.09)	3.02 (1.93–4.74)	1.88 (0.92–3.85)
Psychiatric patient care	1.88 (1.62–2.17)	1.68 (1.45–1.95)	3.68 (2.92–4.65)	2.90 (1.93–4.36)
No psychiatric care for a mental disorder before 2007				
Sickness absence	1.34 (1.15–1.58)	1.20 (1.02–1.40)	0.91 (0.69–1.21)	0.81 (0.50–1.30)
Disability pension	1.78 (1.01–3.14)	1.25 (0.71–2.22)	2.31 (1.19–4.46)	0.68 (0.01–4.84)
Psychiatric patient care	2.67 (2.01–3.54)	1.81 (1.36–2.41)	1.27 (0.80–2.02)	0.96 (0.43–2.14)

a Model 0: crude.

b Model 1: adjusted for sex, age, level of education, area of residence, and family situation; sickness absence, unemployment, inpatient care or specialized outpatient care for common mental disorder, and musculoskeletal disorder between 2004 and 2006; psychiatric medication prescribed between 2005 and 2006.

## Discussion

In this prospective cohort study with 65 097 twin individuals, we aimed to investigate if suicide attempt was associated with future sickness absence, disability pension and psychiatric patient care and if familial factors (genetics and early, shared environment) and previous psychiatric care play a role in the associations. The results of this study showed that there were significant associations between attempted suicide and subsequent work incapacity/psychiatric patient care.

This was as expected given the close connection between suicidal behavior and poor mental health (Hawton & van Heeringen, 2009). However, our results were controlled also for previous common mental disorders and prescribed psychiatric medication, which might have underlined the association between suicide attempt and mental health. Furthermore, results of the analyses accounting the baseline mental health status (i.e. those who had received psychiatric care before 2007 and those who had not) did not show a substantial difference in HRs. Only in DZ twins, individuals who had received previous psychiatric care had a somewhat higher risk of future mental disorders compared to those without previous psychiatric care.

The results of this study suggest that there might be unrecovered or undetected underlying mental disorders of individuals who are known to have attempted suicide, since they still have a higher risk for future mental diagnoses after being treated. Previous research suggests that only around 30% of individuals with mental disorders visit health care (Appleby et al., 2017) and thus it is likely that unrecognized mental disorder underlies the association of suicide attempt and future mental disorders. The finding may also signal that the treatment of mental disorders is not effective enough among individuals who are known to have attempted suicide, since they still have a higher risk for future mental disorders after being treated. While this might at first read as a critique of the current health care system, it may just as well be a testament to the recalcitrant nature of many mental disorders. Therefore, the finding indicates that there is a need of designing measurements and treatments to detect and treat mental disorders and suicidal behavior more effectively. Moreover, having attempted suicide was also associated with a 1.24 times increased risk for subsequent sickness absence, and a 1.81 times increased risk for disability pension, which is in line with three Swedish prospective cohort studies by using population-based data (Niederkrotenthaler et al., 2018, 2014, 2017).

The risk of attempting suicide for those with sickness absence or disability pension diluted in the conditional analysis. However, among MZ twins the association between attempted suicide and subsequent psychiatric patient care reached significance. Similar results were found in the analyses stratified by previous psychiatric care, suggesting that familial factors influence the association between attempting suicide and subsequent work incapacity. It is possible that mental or physical ill-health due to genetic susceptibility, personality, or socioeconomic status which are known to be affected by genetics or shared environment may have an impact on the associations between suicide attempt and work incapacity (Hettema et al., 2001).

### Methodological considerations

The present study was a population-based twin study, i.e. including comprehensive register data without loss to follow-up from 2007 to 2013. The strengths of this approach are the high number of individuals included, giving the study enough power to establish statistical associations with high certainty. The register data used in this study, including data from the Swedish Twin Registry, the national inpatient register, and LISA, are also considered to be of high quality, with minimal selection bias and generally high validity (Lichtenstein et al., 2002; Ludvigsson et al., 2011; Ludvigsson, Svedberg, Olen, Bruze, & Neovius, 2019). This of course helps in producing reliable results. A twin study design was chosen, as it is a natural experiment design which can control for unmeasured familial factors.

Limitations include that the study only included data for sickness absence that lasted 14 days or longer since the Social Insurance Agency does not gather data for shorter periods. This may have led to an underestimation of the effect of exposure on sickness absence. Moreover, data on attempted suicide, along with several other healthcare-related variables, was also solely based on inpatient care and specialized outpatient care. Because of this, the present study could not gather data from other forms of care such as primary care, nor those that did not seek or otherwise acquire any help for their disorders. Also, data availability varies in different registers. For instance, there were no available data on psychiatric medication before 2005 while data on specialized outpatient care were not available before 2001, which might cause over- or underestimation of the results. However, this effect is possible to be non-differential in MZ and DZ twins and our estimation of the influence of familial factors might not be biased. Despite these, the results ought to generalize well to culturally and developmentally comparable societies with similar social insurance systems, at least in Nordic countries.

## Conclusions

Attempting suicide is a predictor of subsequent work incapacity and psychiatric patient care. Familial factors (genetics and shared environment) and previous psychiatric care played a role in these associations. Hence the effects of familial factors and previous patient care for mental diagnoses should be accounted for work incapacity and psychiatric patient care after a suicide attempt, and perhaps the specific diagnoses that lead to work incapacity could be elaborated.
